# Gregory (Greg) Stores, MD, FRCP, FRCPsych

**DOI:** 10.1192/bjb.2023.35

**Published:** 2023-12

**Authors:** Rachel Stores

Formerly Professor of Developmental Neuropsychiatry, University of Oxford, UK

**Figure:**
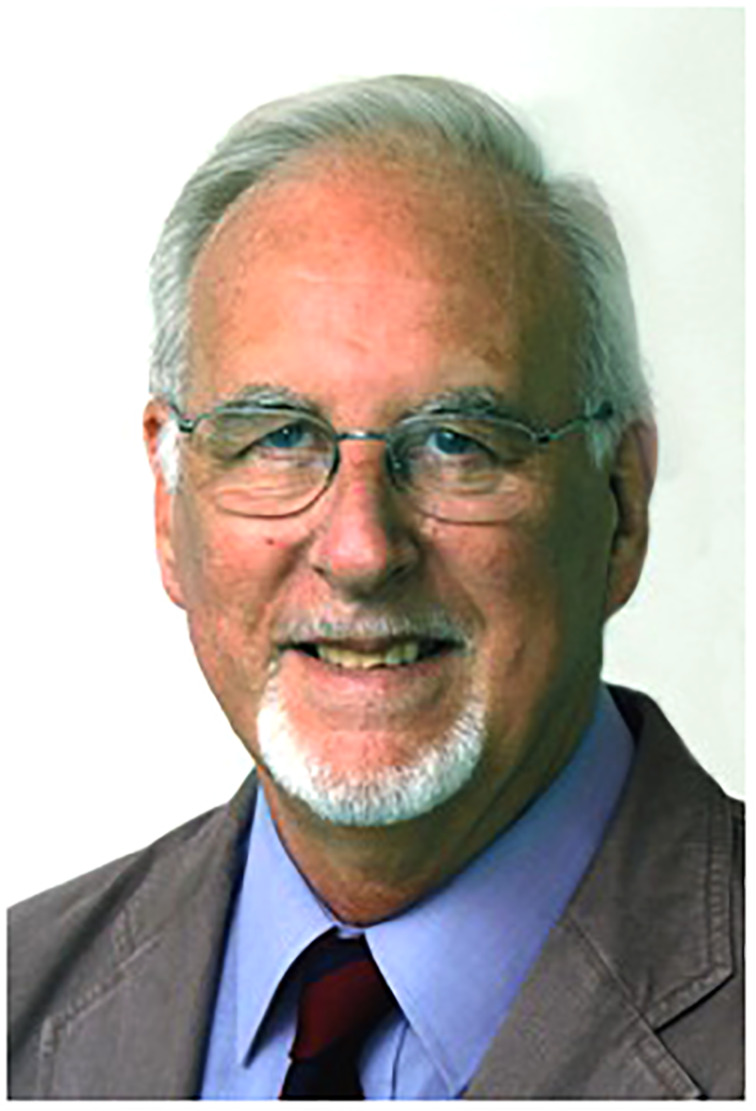

Greg Stores, who died on 21 December 2022 aged 84, throughout his career contributed greatly to the understanding of epilepsy and sleep disorders in children. Working as Director for the Oxford Regional Paediatric EEG Service he developed a number of successful research programmes, securing a research grant total in the region of £1 m principally in support of studies in children's sleep disorders. One of his most significant contributions to the diagnosis of childhood epilepsy was his introduction of ambulatory EEG in real-life settings. This technique has become the worldwide standard for research and clinical investigation of epilepsy and sleep disorders in patients of all ages. He authored many highly cited academic journal articles and was an author and editor of several books, including *A Clinical Guide to Sleep Disorders in Children and Adolescents* and *Sleep and Its Disorders in Children and Adolescents with a Neurodevelopmental Disorder*.

Greg was born in Stockport in 1938, as the only child of parents William, a labourer, and Marion, a factory worker. He went to school at St Bede's College, where he became Head Boy, and spent many happy hours of his youth in Stockport Library. After achieving a first class degree in psychology at the University of Manchester, he went on to study medicine there. While excelling at university, he showed his passion for athletics, competing in throwing events including the hammer up to County, Northern Counties and British Universities representative levels.

After graduating in 1967 he worked in various hospitals across the North of England in both medical and psychiatric settings before moving to Oxford. He completed a Visiting Fellowship at Yale University Department of Neurology in 1974 and was appointed a consultant in developmental psychiatry in 1976, working at the Park Hospital in Headington in both sleep disorder and epilepsy services for patients of all ages referred from throughout the UK.

Greg was appointed Professor of Developmental Neuropsychiatry and Fellow of Linacre College at the University of Oxford in 1999. He taught undergraduate and postgraduate groups in medicine and allied disciplines in other centres, including Warwick Medical School. Following his retirement he worked at the Institute of Child Health, University College, London, while also continuing to publish articles and books and undertaking medico-legal work as an expert witness. Owing to his international reputation, throughout his career he both organised and was invited to be a keynote speaker at conferences and scientific meetings around the world. He also made national and international television and radio appearances and press contributions.

Alongside his extensive knowledge, achievements and clinical capabilities, Greg was known as a kind and generous doctor, colleague and friend. Many who have worked with him have shared how much they learned from his way of teaching and sharing knowledge, and how his support and guidance have greatly influenced their careers. His approachability and warmth were coupled with integrity, refusing to compromise on matters he believed in strongly. He was also known for his sense of humour and fun, and his enthusiasm for a party both at work and at home.

Outside work, Greg had many interests, including a long-standing love of local and social history. He had a particular interest in 17th century and earlier oak furniture, carvings and related items and had an eye for quality pieces. This was evidenced by his fine collection, suitably housed in a beautiful early 17th century house of which he was very proud.

After retiring from clinical work, Greg completed a Postgraduate Certificate in History of Medicine at Oxford Brookes University and was an active and highly valued member of the Dorchester on Thames Historical Society. He meticulously researched and presented on topics as varied as ‘Maladies of Medieval Monks’, ‘The History and Folklore of European Witchcraft and Attempts to Combat Its Influence’ and ‘Charles Dickens as Social Reformer and Medical Observer’. Having lived in Dorchester for almost 40 years he was a significant presence in the community, contributing to it in many ways, including as an Abbey steward, regularly taking visiting groups on historical tours of the building. He helped wash dishes at the village volunteer-run charity tearoom. On his passing, it was in Dorchester Abbey that a large number of friends, family and colleagues met in a ceremony to celebrate his life.

He will be greatly missed by his wife Christina, their two children Rachel and Alasdair, a son Adrian and a daughter Rebecca from his first marriage to Olga, as well as four grandchildren, Beth, Abby, Kaya and Erin.

